# Valorization of *Gleditsia triacanthos* Invasive Plant Cellulose Microfibers and Phenolic Compounds for Obtaining Multi-Functional Wound Dressings with Antimicrobial and Antioxidant Properties

**DOI:** 10.3390/ijms22010033

**Published:** 2020-12-22

**Authors:** Ioana Cristina Marinas, Eliza Oprea, Elisabeta-Irina Geana, Oana Tutunaru, Gratiela Gradisteanu Pircalabioru, Irina Zgura, Mariana Carmen Chifiriuc

**Affiliations:** 1 Research Institute of the University of Bucharest-ICUB, Microbiology Department, Faculty of Biology, University of Bucharest, 91-95 Spl. Independentei, 050095 Bucharest, Romania; ioana.cristina.marinas@gmail.com (I.C.M.); gratiela87@gmail.com (G.G.P.); carmen.chifiriuc@gmail.com (M.C.C.); 2 National Institute of Research & Development for Food Bioresources—IBA Bucharest, 6 Dinu Vintila Street, 021102 Bucharest, Romania; 3 Department of Organic Chemistry, Biochemistry and Catalysis, Faculty of Chemistry, University of Bucharest, 4-12 Regina Elisabeta, 030018 Bucharest, Romania; 4 National R&D Institute for Cryogenics and Isotopic Technologies—ICIT Rm. Valcea, 4 Uzinei Street, PO Raureni, 240050 Ramnicu Valcea, Romania; irina.geana@icsi.ro; 5 National Institute for Research and Development in Microtechnologies IMT-Bucharest, Erou Iancu Nicolae Street, 126A, 077190 Bucharest, Romania; oana.tutunaru@imt.ro; 6 Department of Optical Processes in Nanostructured Materials, National Institute of Materials Physics Atomistilor Street, 405A, 077125 Magurele, Romania; irina.zgura@infim.ro

**Keywords:** multi-functionalized cellulose microfibers, controlled release, wound dressing, antimicrobial activity, antioxidant activity

## Abstract

*Gleditsia triacanthos* is an aggressive invasive species in Eastern Europe, producing a significant number of pods that could represent an inexhaustible resource of raw material for various applications. The aim of this study was to extract cellulose from the *Gleditsia triacanthos* pods, characterize it by spectrophotometric and UHPLC–DAD-ESI/MS analysis, and use it to fabricate a wound dressing that is multi-functionalized with phenolic compounds extracted from the leaves of the same species. The obtained cellulose microfibers (CM) were functionalized, lyophilized, and characterized by ATR-FTIR and SEM. The water absorption and retention capacity as well as the controlled release of phenolic compounds with antioxidant properties evaluated in temporal dynamics were also determined. The antimicrobial activity against reference and clinical multi-drug-resistant *Escherichia coli, Staphylococcus aureus, Pseudomonas aeruginosa, Acinetobacter baumannii, Enterobacter cloacae, Candida albicans,* and *Candida parapsilosis* strains occurred immediately after the contact with the tested materials and was maintained for 24 h for all tested microbial strains. In conclusion, the multi-functionalized cellulose microfibers (MFCM) obtained from the reproductive organs of an invasive species can represent a promising alternative for the development of functional wound dressings with antioxidant and antimicrobial activity, as well as being a scalable example for designing cost-effective, circular bio-economy approaches to combat the accelerated spread of invasive species.

## 1. Introduction

Micro- and nanomaterials derived from biological macromolecules are used in a wide range of industrial, technological, and biomedical applications, e.g., for adsorption, ultrafiltration, packaging, preservation of historical artefacts, thermal insulators, and fire retardants, energy extraction and storage, acoustics, sensory, controlled delivery of drugs, and especially tissue engineering. Due to their mechanical robustness, hydrophilicity, biocompatibility, and good biodegradability, micro- and nanocellulose have specific functions, such as tissue repair, regeneration, and healing, and they are also antimicrobial nanomaterials with shape memory and intelligent membranes [1,2]. 

Different types of cellulose with a well-defined and functionalized morphology with different active principles can be used to obtain advanced wound dressings with high performance for preventing wound infections and accelerated wound healing [1]. Previous studies of wood-based nanofibrillated cellulose (NFC) have shown a high capacity to absorb liquids and form translucent films, suggesting the benefits of using cellulose fibers as a dressing. The property of maintaining a moist environment is necessary for the proper healing of chronic wounds, while translucency makes it possible to evaluate the wound progress without requiring the dressing removal [3]. In addition, these nanomaterials can provide various drug delivery properties and other effects, depending on the raw materials they come from [4] and can be used for the design of functional wound dressing or for topical drug delivery, offering several advantages over other administration pathways, including eliminating internal metabolism, minimizing pain, prolonging the drug release, and controlling drug absorption by removing skin bandage [5]. On the other side, Nie (2019) showed that the nanocellulose fibers (NF) have lower thermal stability compared with raw microcellulose fibers (MF) due to the reduced particle size, increased specific surface area, and increased exposed carbon and reactive group activity. The thermal degradation of NF generates low molecular weight segments and breakpoint defects. The same effect was produced after introducing the carboxylate group on the cellulose surface of the nano-sized fiber (prepared by oxidation of TEMPO ((2,2,6,6-tetramethyl-1-piperidinyl)oxidanyl) [2]. Microcellulose fibers can be obtained from different plant species (including invasive ones), such as bamboo [6], *Artemisia vulgaris* [2], and banana plant wastes [7]. 

*Gleditsia triacanthos* L. (*Fabaceae*) is a native tree to North America, with an extraordinary tolerance to high temperatures and drought, conditions that paradoxically favor its spread. This species is also very tolerant of industrial and transport emissions with relatively high resistance to soils with extreme pH. The combined clonal and sexual reproduction, the short juvenile period, the high seed production and the high germination rate of the seeds are considered defining traits for the degree of invasiveness of these species [8]. Initially introduced as an ornamental and aromatic species, it spread rapidly, invading different ecoregions, being presently considered one of the most aggressive legume invaders in the world [9]. This species invasiveness poses serious risks for ecosystems biodiversity, structure, and functions, as well as conservation of the protected areas, and requires high costs for its eradication [10]. The selective harvesting of reproductive organs of such invasive species for practical use could stop their uncontrolled spread. Taking into account that plant extracts offer unlimited therapeutic options, one of the sectors that could benefit from the potential use of invasive plants is the biomedical field.

Thus, the therapeutic valorization of these species could be both an ecological solution to stop their uncontrolled spreading and an economic solution, providing low costs raw materials. The purpose of the study was to obtain cellulose microfibers (CM) from the reproductive organs of *Gleditsia triachantos* and functionalize them with active principles (phenolic compounds from *G. triachantos* leaves) with antioxidant and antimicrobial activity in order to obtain functional wound dressings with improved performance in preventing wound infections, therefore, accelerating the wound healing process. 

## 2. Results

### 2.1. Chemical Characterization of Phenolic Compounds of G. triachantos Leaves 

#### 2.1.1. Total Phenolic Compounds (TPC)

The quantification of total phenolic compounds (TPC) was performed using the calibration curve, resulting in a content of 113.62 ± 1.09 mg TPC (GAE, gallic acid equivalents)/g dry plant, and the flavonoid content was 35.24 ± 0.55 mg (QE, quercetin equivalents)/g dry plant for the *G. triachantos* leaves extract.

#### 2.1.2. Ultra-High-Performance Liquid Chromatography Diode Array Detector Electrospray Ionization Tandem Mass Spectrometry (UHPLC–DAD-ESI/MS)

Phenolic compound profile by UHPLC–DAD-ESI/MS (ultra-high-performance liquid chromatography diode array detector electrospray ionization tandem mass spectrometry). The concentrations of phenolic acids (PAs), flavonoids, and flavonoid heterosides are shown in Table S1 of Supplementary Materials. 

The main phenolic compounds identified in the alcoholic extract of G. triachantos were vanillic acid (773.88 µg/L), 4-hydroxy benzoic acid (570.37 µg/L), ferulic acid (319.81 µg/L), catechin (34,271.15 µg/L), quercetin (3,007.89 µg/L), and epicatechin (1,431.47 µg/L). The extract was rich in chlorogenic acid (11,984.43 µg/L), cinnamic acid (2,736.42 µg/L, an acid that lacks phenolic groups but is still usually included in the phenolic compounds class), and rutin (1,302.31 µg/L). The following compounds were also evidenced by UHPLC–DAD-ESI/MS: abscisic acid (16.10 µg/L), a sesquiterpenoid plant hormone, and ellagic acid (165.13 µg/L), a dimer of gallic acid. 

### 2.2. Multi-Functionalized Cellulose Microfiber Fabrication 

*G. triachantos* pods were ground into powder (to pass through a 100 mesh sieve) with a structure characterized by ATR-FTIR (Attenuated Total Reflectance—Fourier Transform Infrared) to highlight the specific bands of the three major components (see Section 2.3.1), which are hemi-cellulose (39.07% ± 1.49%), cellulose (29.78% ± 1.28%), and lignin (27.71% ± 0.75%) [11]. Then, the wood powder was subjected to purification by successive extractions to remove the extractable substances, hemicellulose (toluene and ethanol), and lignin. The lignocellulosic material required six cycles of delignification. The sample was taken from each cycle to quantify the total lignin content by a similar protocol to that used to determine the hydroxycinnamic acid content. The delignification process was continued for two more cycles after the lignin content was below the detection limit. After the last delignification cycle, the aqueous suspension of the obtained cellulose fibers was washed until no chloride ions were found, quantified by the Mohr titrimetric method. After extraction, the freeze-dried cellulosic material was characterized to highlight the lack of lignin content and compared to CM functionalized with phenolic compounds extracted from the leaves of the same species.

### 2.3. Characterization of Cellulose Microfibers (CM) and Multi-Functionalized Cellulose Microfibers (MFCM)

#### 2.3.1. ATR-FTIR Analysis

**Figure 1:**
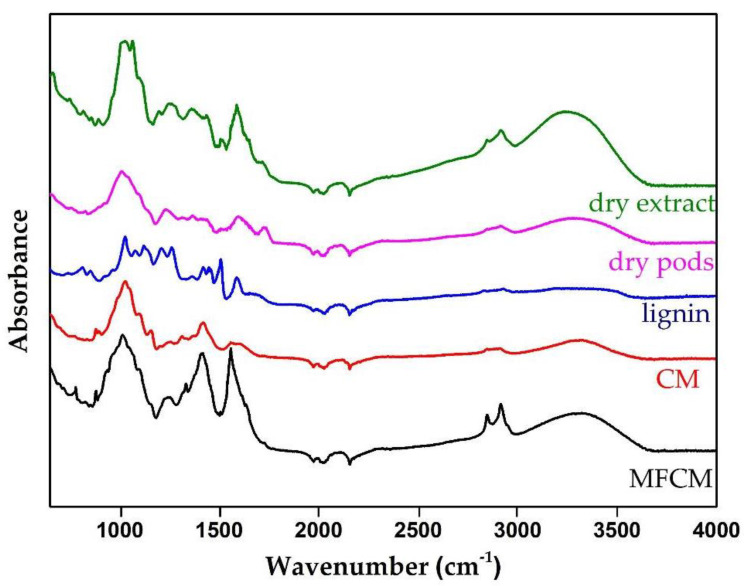
Attenuated Total Reflectance—Fourier Transform Infrared (ATR-FTIR) spectra of multi-functionalized cellulose microfibers (MFCM) (black line), CM (red line), lignin (blue line), initial dry pods (pink line), and dry extract (green line).

The ATR-FTIR spectra of the studied materials, CM and multi-functionalized cellulose microfibers (MFCM), are presented in Figure 1. According to Danial (2015) [12], the lack of the C=O band in both spectra that should have been observed at the wave number ≈1733 cm^−1^ highlights the successful removal of hemicelluloses. The complete removal of lignin was given by the lack of bands at ≈1509 cm^−1^ and ≈1592 cm^−1^, bands attributed to the aromatic C=C vibrations in the plane as well as the lack of the band located at 1264 cm^−1^, which would have been assigned C–O–C specific to ether bonds from lignin. The C–C band breathing rings (≈1158 cm^−1^) and the C–O–C glycosidic ether band (≈1100 cm^−1^) were attributed to the presence of cellulose. The band at ≈2900 cm^−1^, observed in all samples, corresponded to the C–H stretching vibration that reflects the organic content in general [13]. 

From the literature data, it is known that the bands in the region 1680–900 cm^−1^ may have come from phenolic compounds specific to the extract [14,15].

Among others, several specific aromatic C–H vibrations were present between the wavenumbers 670–900 cm^−1^ and 950–1225 cm^−1^ [16]. The band at the level of 1247 cm^−1^ corresponded to the extension of pyran rings, typical for flavonoid compounds [17]. 

Theoretically, the cellulose–PAs and cellulose–flavonoids bonds are the ester and ether ones, respectively, which were indeed highlighted at the wavenumbers 1338 cm^−1^ and 1260–1230 cm^−1^, respectively. The bands specific to the ester groups were similar to those in the dry extract because PAs are found in the form of glycosidic esters according to Qian et al. [18]. By comparing the FTIR spectra of CM, MFCM, and the dry extract, we identified four specific bands for MFCM that were not found in the other spectra, at the wavenumbers 779 cm^−1^ (aromatic C–H out-of-plane bending), 1338 cm^−1^ (C–O stretching (phenyl), C–H (bending and CH_2_), 1442 cm^−1^ (C–C stretching vibration in aromatic ring), and 1558 cm^−1^ (C=C–C aromatic ring stretch) [19].

#### 2.3.2. Morphology of CM and MFCM

**Figure 2:**
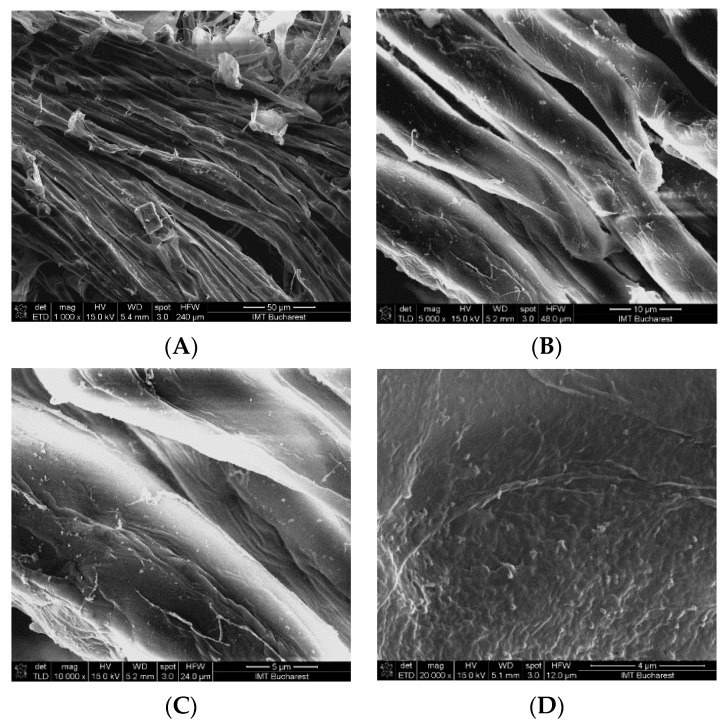
SEM morphology of the reference CM surface obtained from *Gleditsia triacanthos* pods, used for functionalization with active principles from *G. triacanthos* leaves: (**A**) 1000×, (**B**) 5000×, (**C**) 10,000×, and (**D**) 20,000×.

**Figure 3:**
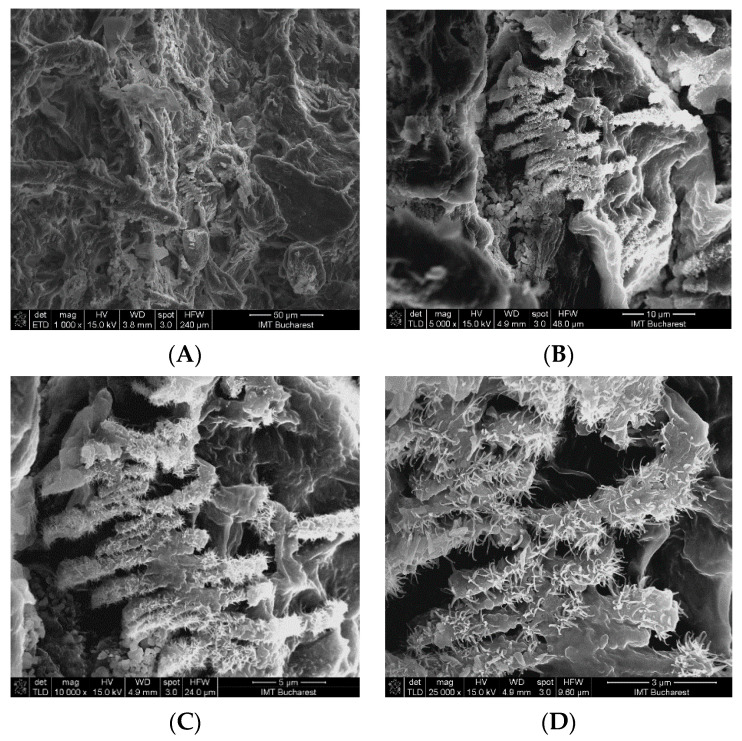
SEM morphology of the MFCM (multi-functionalized cellulose microfiber): (**A**) 1000×, (**B**) 5000×, (**C**) 10,000×, and (**D**) 20,000×.

SEM (scanning electron microscopy) analysis revealed that the control sample (CM) used for multi-functionalization with phenolic compounds showed the presence of microfibers (Figure 2) with diameters between 5 and 10 μm. The surface morphology of the CM sample indicated a relatively smooth surface, while that of the MFCM highlighted a rougher surface due to the adherence of the phenolic compounds. In the case of MFCM (impregnated with the extract from *G. triachantos* leaves), it can be observed from Figure 3 that the chemical components specific to the extract adhered to the cellulose microfiber surface without requiring the presence of a fixing or crosslinking agent. These components (of MFCM) had a thickness between 40 and 120 nm and a length 0.2–1 μm. 

#### 2.3.3. Surface Wettability 

The CA (contact angle) measurements revealed that CM and MFCM samples showed a superhydrophilic behavior (CA ≈ 0°), with the water drops being absorbed in the sample volume (Video Supplementary Materials).

#### 2.3.4. Water Absorptivity and Retention Properties

The percentage of PBS (phosphate-buffered saline) uptake was slightly improved from 674.4% for the CM (control sample) to 721.51% for the MFCM. The freeze-dried phenolic compounds adhered to microfibers could be responsible for the increased absorption capacity, most probably due to the presence of the hydroxyl groups. 

**Figure 4:**
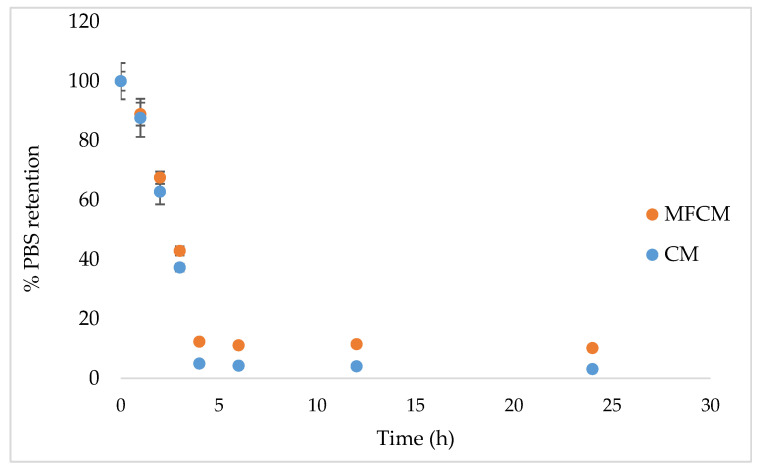
The water retention capacity of CM and MFCM matrices over time (*p* < 0.05).

The difference in terms of water retention capacity (PBS) between the CM (control) and MFCM remained constant after 4 h, however, MFCM had better water retention properties (Figure 4). 

#### 2.3.5. In Vitro Phenolic Compound Release Studies and Antioxidant Activity 

**Figure 5:**
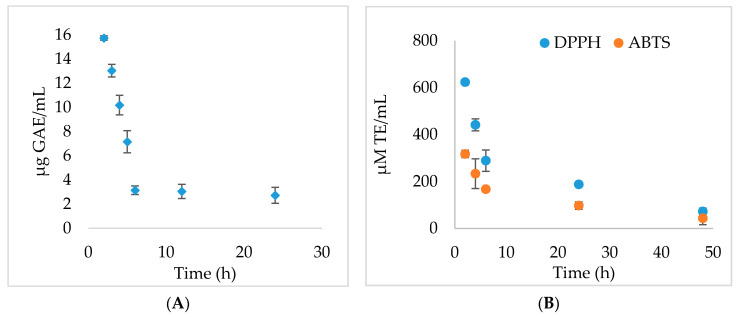
Kinetics of controlled release of phenolic compounds (**A**) and antioxidant effects (**B**) in temporal dynamics for multi-functionalized cellulose microfibers (MFCM), *p* < 0.05.

The release kinetics of the active principles were evaluated by determining the TPC content by the Folin–Ciocalteu method and through monitoring the antioxidant activity over time. The process of releasing the phenolic compounds from the MFCM varied considerably in time (Figure 5A)—in the first 6 h, the release was linear (*R*^2^ = 0.9935), with a velocity of 3.1083 μg GAE/mL * h. The release of the phenolic compounds from the cellulose-based dressings was relatively short, reaching an almost complete release of the extract from MFCM within 120 h. A sudden release of a high concentration of TPC in PBS was observed after 2 h, with the rest being released in inverse proportion to the contact time. All experiments were performed in triplicate and the results have statistical significance (*p* < 0.05).

The TEAC (Trolox equivalent antioxidant capacity) results have shown that the highest concentration of compounds with antioxidant potential was released in the first 6 h [20], while in the DPPH (1,1-diphenyl-2-picrylhydrazyl radical) assay, the antioxidant activity was inversely proportional to the contact time, observing a constant release until the complete reduction of the phenolic compound content in the material (Figure 5B).

### 2.4. Antimicrobial Activity

The antimicrobial activity was performed by evaluating the viable cell counts (colony forming unit (CFU)/mL) and calculating the recovery factor initially and after 24 h. The data from Table 1 show that the active principles released from the cellulose matrix positively influenced the antimicrobial effect both compared to the strain and CM control. The MFCM showed a better antimicrobial activity than CM for all strains except *Enterococcus faecium* ATCC DMS 13590 after 24 h of incubation at 37 °C.

### 2.5. Assessment of Cells Viability and Cytotoxicity 

**Figure 6:**
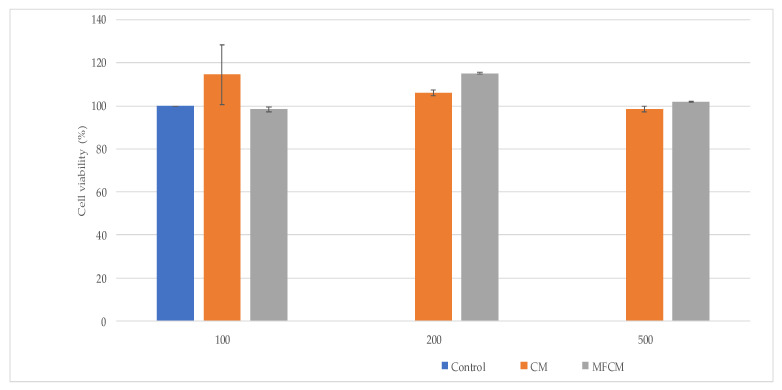
Cell viability (%) investigated by MTT ([3-(4,5-dimethylthiazol-2yl)]-2,5-diphenyltetrazolium bromide) assay—L929 cells co-cultured with CM and MFCM at various concentrations: 100, 200, and 500 µg/µL.

**Figure 7:**
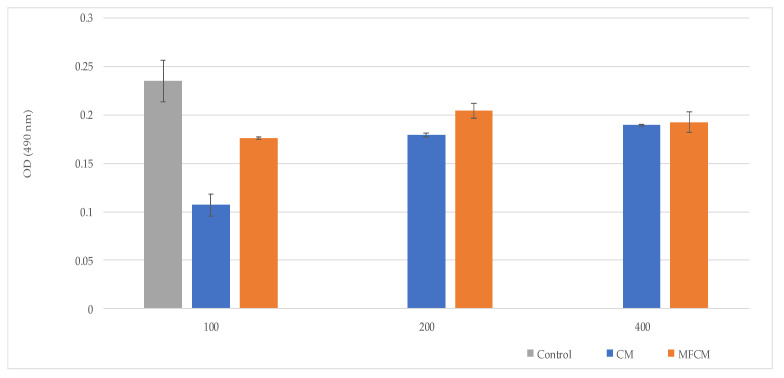
Cell cytotoxicity measured by LDH (lactate dehydrogenase) assay—L929 cells co-cultured with CM and MFCM at various concentrations: 100, 200, and 500 µg/µL.

Cell viability and cell cytotoxicity were investigated by MTT ([3-(4,5-dimethylthiazol-2yl)]-2,5-diphenyltetrazolium bromide) assay—L929 cells, and by LDH (lactate dehydrogenase) assay—L929 cells, respectively, co-cultured with CM and MFCM (Figure 6 and Figure 7). 

Among the tested compounds, CM (100 µg/uL) harbored the highest level of biocompatibility with high cell proliferation rates and low LDH.

The high rates of cell viability in contact with the CM and MFCM demonstrated that these new cellulose microfibers obtained from *Gleditsia triachantos* are biocompatible materials.

## 3. Discussion

Phenolic compounds are plant secondary metabolites with a strong antioxidant effect, being known to initiate numerous processes involved in the wound healing process [21,22]. In this study, we aimed to extract the cellulosic material from the *G. triacanthos* pods, which was further multi-functionalized with a leaf extract of the same species, showing a significantly highphenolic compound content that in a first stage could exhibit both antimicrobial and antioxidant activities, contributing to skin regeneration. Data regarding UHPLC-DAD-ESI/MS analysis of PA and flavonoids from *G. triachantos* leaf extract have not been previously reported. However, Mohammed (2014) isolated and identified eight flavone glycosides and two flavone aglycones such as luteolin-7-O-β-glucopyranosid, luteolin-7-O-β-galactopyranoside, apigenin-7-O-β-glucopyranoside, luteolin, and apigenin from the aqueous ethanol extract of *G. triacanthos* L. leaves. Vitexin, luteolin, isovitexin, and quercetin have also been previously identified in this plant extract [23]. In addition, some identified compounds from the alcoholic extract of *G. triachantos*, such as syringic acid [24], gallic acid [25], p-coumaric acid [26], apigenin [27], quercetin [28], and myricetin [29], were reported to exhibit anti-inflammatory effects. Thus, PAs promise to be multivalent, bioinspired molecules that either alone or in the form of biomaterials additives can be very useful to prevent and treat medical device-associated and drug-resistant infections [30].

On the other hand, Saleh (2016) [31] reported the analgesic activity of the fruit methanolic extract of *G. triacanthos* and the saponins fraction derived, but the presence of these compounds could not be considered in our paper because the foaming test (for saponins) was negative.

To the best of our knowledge, the microcellulose obtained from *G. triacanthos* pods is herein reported for the first time, although some studies have been carried out on the characterization of other polysaccharides such as galactomannan [32], which is a heterogenous, water-soluble, non-toxic, and biodegradable polysaccharide used as a stabilizing, thickening, and emulsifying agent in the biomedical industry. The cellulose was extracted by treatment of grounded pods with toluene/ethanol (2:1) solution and six cycles of delignification method adapted after Zhuo (2017) [33]. The cellulose obtained was divided quantitatively into two parts—one was treated with 50% ethanol (control) in accordance with the second part, which contained the extract from the leaves of the same species. After lyophilization, the two materials were evaluated by ATR-FTIR analysis and compared to the commercial lignin. SEM analysis revealed that the control sample (CM) used for multi-functionalization with phenolic compounds showed the presence of microfibers and that the extract adhered to the cellulose microfibers surface, without requiring the presence of a fixing or crosslinking agent. The data were in accordance with Khalil (2020) [34], which concluded that by acid hydrolysis, the fiber size was in the range of 9–16 μm. In the wound dressing case, it was shown that cellulose microfibers improved the hemostasis of the scaffolds without affecting its cytotoxicity and the strength and flexibility of the films, which could be used in drug delivery or active wound dressing.

SEM (scanning electron microscopy) analysis revealed that the presence of microfibers with diameters between 5 and 10 μm and CM sample indicated a relatively smooth surface while the MFCM surface morphology highlighted a rougher surface compared to the CM due to the adherence of the phenolic compounds.

Microscopic characteristics, such as surface roughness, surface energy of materials, and thin surface coatings, play an important role in determining the wettability of material as well as hydrophobicity/hydrophilicity characteristics. The CM and MFCM highlighted a high wettability surface by CA measurements. The high wettability surface could be attributed to hydroxyl groups located at the equatorial positions of glucopyranose rings corresponding to higher planar orientation. [35]. On the other hand, it is well known that hydrophilicity is one of the most important surface characteristics of wound dressings. The water absorption study is a gravimetric test that has the main purpose of determining the maximum amount of water absorption and retention expressed in percentages. This assay was designed to determine how a dressing manifests its properties in conditions as close as possible to what can happen on the surface of a wound. In essence, the weight gain of the dressing after the absorption of the liquid and its swelling over a certain period of time was measured and used as an indication of water absorption and retention. MFCM had better water retention properties than CM, possibly due to the presence of phenolic compounds and glycosides. In general, these compounds increased the rate of hydration, which represents an advantage for wound dressing materials.

It was observed that MFCM had a statistically significant better retention capacity than CM, which means that it will be more helpful for wounds with moderate exudate and for dry wounds. Water loss could allow exudate and edema fluid to be picked up from wounds in the dressing through an active process directed upwards, as has been reported for some commercially available dressings [36,37]. 

Another important aspect for MFCM is the modulation of antioxidant activity during the release of active principles from the material. Thus, in wound healing, in addition to the antimicrobial potential of dressings, an important aspect is the modulation of reactive oxygen species (ROS) release. It is well known that low levels of ROS can exhibit positive effects, including an antimicrobial effect, but their excessive production can lead to oxidative stress, which will further inhibit the wound healing process, thus favoring wound chronicity [38]. In addition, the plant polyphenols inhibit the release of pro-inflammatory mediators and have no toxicity effects on human tissues [39]. 

There are many studies that have tried to establish some correlations between the antioxidant and antimicrobial activity, but only a few involved clinical strains isolated from hospital-acquired wound infections, such as *Escherichia coli* and *Staphylococcus aureus*, although the results were, however, contradictory [30]. MRSA and *E. coli* seem to be sensitive to cinnamic acid [40], p-coumaric acid [41,42], caffeic acid, vanillic acid, protocatechuic acid [41,43], while ferulic acid was effective only against *E. coli* [43]. On the contrary, in other studies, *E. coli* was not sensitive to p-coumaric acid, vanillic acid, or proocatechuic acid [44]. The MFCM proved to be more active on Gram-negative strains. Previous studies have shown only a minimal link between the antioxidant activity and antimicrobial efficacy [30]. In addition, the CM and MFCM are biocompatible materials with high cell proliferation rates and low cytotoxicity on mouse murine fibroblast cells.

## 4. Materials and Methods

### 4.1. Plant Material

*G. triachantos* leaves and shells were collected from Ramnicu Valcea, Romania, in September 2019 from spontaneous flora. Their taxonomic affiliation was confirmed, and voucher specimens were deposited in the herbarium of the Botanical Garden “Dimitrie Brândză” from the University of Bucharest (no. 40065). The plant materials were manually sorted and dried at room temperature. 

### 4.2. Phenolic Compounds Extraction from G. triachantos Leaves and Chemical Characterization

#### 4.2.1. Hydro-Alcoholic Extraction from *G. triachantos* Leaves

An amount of 2.5 g of previously ground dry leaves was weighed and brought into 50 mL of ethanol solution 50%. The extract obtained by the ultrasound-assisted extraction method was maintained at room temperature for 20 days, and after that, it was sonicated again for 30 min. Then, it was filtered and stored at −20 °C until it was incorporated in cellulose microfibers.

#### 4.2.2. Total Phenolic Compounds and Flavonoids Contents 

TPC content was determined by the Folin–Ciocalteu method [45]. An aliquot was mixed with 0.1 mL Folin–Ciocalteu reagent, 1.8 mL distilled water, and 0.1 mL of saturated sodium carbonate. The tubes were vortexed for 15 s and allowed to stand at dark for 60 min for color development. Absorbance was then measured at 765 nm using a FlexStation 3 UV–VIS (Molecular Devices Company, Sunnyvale, CA, USA) spectrophotometer. A standard curve was prepared by using different concentrations of gallic acid in the same condition with samples (*R*^2^ = 0.9985). TPC content was expressed as milligram gallic acid equivalent/g dry leaves (mg GAE/g DL). Analyses were performed in triplicate.

Flavonoid content was assessed through the AlCl_3_ method [46]. Briefly, 0.1 mL sample/standard solution was mixed with 0.1 mL sodium acetate 10% and 0.12 mL AlCl_3_ 2.5%, with the final volume being adjusted to 1 mL with ethanol 50%. The samples were then vortexed and incubated in the dark for 45 min. The absorbances were measured at λ = 430 nm. A standard curve was prepared by using different concentrations of quercetin (*R*^2^ = 0.9958). Total flavonoid content was expressed as milligram quercetin equivalent/g dry leaves (mg QE/g DL). Analyses were performed in triplicate.

#### 4.2.3. Ultra-High-Performance Liquid Chromatography Diode Array Detector Electrospray Ionization Tandem Mass Spectrometry

All experiments were performed using an UltiMate 3000 UHPLC system (ThermoFisher Scientific, Bremen, Germany), consisting of a quaternary pump, diode array detector (DAD) set at 280 nm, column oven, and autosampler coupled to a Q Exactive Focus Hybrid Quadrupole-OrbiTrap mass spectrometer equipped with heated electrospray ionisation (HESI) probe (ThermoFisher Scientific, Bremen, Germany).

Separations were performed on Kinetex (C18, 100 × 2.1 mm, 1.7 μm, Phenomenex, California, USA) column (reverse-phase UHPLC column) and a binary solvent system consisting of solvent A (water with 0.1% formic acid) and solvent B (methanol with 0.1% formic acid). The UHPLC gradient for mass screening, the flow rate, and the operation conditions of the mass spectrometer were previously optimized [47]. PAs and flavonoids from the extract were also identified and quantified according to Ciucure [47] (Table S1), as well as data acquisition and data analysis.

### 4.3. CM and MFCM Obtaining and Characterization

#### 4.3.1. CM and MFCM Obtaining 

Cellulose extraction was performed according to Zhuo (2017) [33]. Briefly, an amount of 30 g ground shells of *G. triacanthos* was added over toluene/ethanol solution (2:1, *v*/*v*) and the mixture was further refluxed for 8 h at 90 °C. Then, the biomass was treated with sodium hypochlorite under the acidic condition (glacial acetic acid) at 70 °C for 6 h to remove the lignin, followed by treatment with 2% NaOH aqueous solution at 90 °C for 2 h to remove most of the hemicellulose. After that, the samples were again treated by sodium hypochlorite under acidic condition for 2 h, followed by final purification of 2% NaOH solution at 90 °C for 2 h. After each cycle, the presence of lignin was evaluated by hydroxycinnamic acid content according to British Pharmacopoeia [48] using a calibration curve with commercial lignin solubilized in DMSO (dimethylsulfoxide) (λ = 524 nm, *R*^2^ = 0.9996). When lignin content was out of the detection limit, the cellulose was washed until it reached pH = 7.4, and each supernatant was evaluated by titration with AgNO_3_, indicating the removal of chloride ions. The cellulose fibers were divided into 2 samples, one being dispersed in 50% ethanol (control), and the second in the extract obtained previously. Both products were dispersed 3 times using IKA ULTRA-TURRAX (IKA®-Werke GmbH & Co. KG, Staufen, Germany) for 10 min, followed by an ultrasound treatment for 60 min. Finally, the samples were lyophilized at −55 °C and 1 Pa to obtain the cellulose microfibers (CM) and the multi-functionalized cellulose microfibers (MFCM), respectively. 

#### 4.3.2. ATR-FTIR

The FTIR spectra for CM, MFCM, and lignin was recorded at room temperature using the Cary 630 FTIR Spectrometer in ATR mode (Agilent Technologies Inc., Santa Clara, CA, USA) and Agilent MicroLab Software FTIR System (Agilent Technologies, Inc., USA). The chosen measurement range was 4000–650 cm^−1^, number of scans was 400, and resolution was 4 cm^−1^. The FTIR spectra of dry extract were produced on the alcoholic extract of *G. tricanthos* leaves dried previously in an oven at 35 °C (for 24 h).

#### 4.3.3. Morphology of CM and MFCM

The morphology of CM and MFCM were performed by Nova NanoSEM 630 Scanning Electron Microscope (FEI Company, Hillsboro, OR, USA) using UHR detector (Through-Lens-Detector-TLD) at an acceleration voltage of 15 kV and a working distance of about 5 mm. The samples were fixed with a double tape on a conductive support of aluminum, which, afterward, was covered with a few nanometers of gold layer using sputtering for 30 s.

#### 4.3.4. Surface Wettability

Static (equilibrium) CAs were measured using a drop shape analysis instrument, model DSA100 (Krüss GmbH, Hamburg, Germany). The samples were positioned on a plane stage, under the tip of a stainless-steel needle, with a blunt end, with an outer diameter of 0.5 mm. The needle was attached to a syringe controlled by DSA3 software supplied with the instrument and was used to drop water with a controlled volume on the test surface as well as to evaluate the contact angle. The volume of water droplets was ≈1 μL with a pumping rate of 5 μL/min. The tests were carried out at room temperature [49].

#### 4.3.5. Water Absorptivity and Retention Properties

An amount of 0.1 g CM was treated with methanol and dried (60 °C for 24 h), then immersed in PBS (pH 7.4). The excess buffer was removed with filter paper and the material’s wet mass was determined using the method adapted after Ngadaonye (2013) [36]. The same procedure was applied to MFCM, while the percentage of absorbed water was calculated for CM and MFCM as follows:

Swelling ratio (%) = (M_2_ − M_1_) × 100/M_1_

where M_1_ and M_2_ represent the weights of dry and wet samples, respectively. 

Water retention properties were determined using water retention tests. Samples (CM and MFCM) with the same mass were dried and weighed. Samples were then immersed in PBS at 37 °C for 24 h, and the water on the surface was removed using filter paper. Samples were finally placed in plates at 37 °C and weighed after a specific incubation time [50]. The water retention capacity was calculated using the following equation:

Water retention ratio (%) = Mi × 100/M_0_

where M_i_ and M_0_ represent the percent of swelling at specific and initial time (maximum PBS swelling), respectively.

#### 4.3.6. In Vitro Phenolic Compounds Release Studies and Antioxidant Activity

The MFCM were immersed in PBS at 37 °C for 5 days to determine the various compounds’ kinetics release from these dressings. The release studies were conducted in closed glass vessels containing 1 mL PBS. The medium was removed (completely) periodically, at each sampling time (2 h, 4 h, 6 h, 24 h, 48 h, 72 h, 120 h), and fresh medium was introduced. Quantification of the active principle release at different time intervals was determined. The TPC content was determined by the Folin–Ciocalteu method. For each sampling time, the antioxidant activity was determined by DPPH and TEAC (Trolox equivalent antioxidant capacity) methods using a FlexStation 3 UV–VIS spectrophotometer (Molecular Devices Company, Sunnyvale, CA, USA).

The DPPH assay was performed according to the method of Madhu (2013) with some slight changes [51]. The standard curve was linear between 0 and 200 mM Trolox (*R*^2^ = 0.9998). Results were expressed as millimolar TE (Trolox equivalent)/g material.

TEAC assay was performed according to Re (1999) with some modifications [52]. A stable stock solution of ABTS•^+^ (2,2′-azinobis (3-ethylbenzothiazoline-6-sulfonic acid)) was produced by reacting an aqueous solution of 7 mM ABTS with 2.45 mM potassium persulphate. The standard curve was linear between 0 and 200 mM Trolox (*R*^2^ = 0.9978). Results are expressed as millimolar TE/g material.

### 4.4. Antimicrobial Activity

#### 4.4.1. Sterilization of CM and MFCM

The samples were placed in an irradiation container. To measure the treatment doses, we placed dosimeters in the minimum dose positions (D min = 33.2 ± 1.6 kGy) and the maximum dose (D max = 35.7 ± 1.6 kGy). According to the dosimetry calculations performed, the minimum and maximum doses absorbed by the samples were within this range, with a confidence level of 95%. The dosimetry system used was Ethanol-Chlorobenzene (ECB). Gamma irradiation (Co-60) was performed with the SVST Co-60/B irradiator (Institute of Isotopes Co. Ltd. Budapest, Hungary) for 11 h 57 min at a dose ≥25 kGy. For sterility, verification/validation was performed at a concentration of 10% CM in peptone water and the doses were incubated at 37 °C for 24 h. After incubation, a volume of 0.1 mL was inoculated by being displayed on Trypton soy agar and Sabauroud agar in triplicates. The plates were incubated at 37 °C for 24 h for the enumeration of aerobic and mesophillic organisms and 7 days for fungi.

#### 4.4.2. Evaluation of the Antimicrobial Activity

The bacterial strains were *Escherichia coli* ATCC 11229; *Staphylococcus aureus* ATCC 6538; *Pseudomonas aeruginosa* ATCC 27853; *Enterococcus faecium* DMS 13590; and multi-drug-resistant clinical isolates of *Staphylococcus aureus*, *Pseudomonas aeruginosa*, *Acinetobacter baumannii*, and *Enterobacter cloacae* from microbial collection of Microbiology Lab from Faculty of Biology, University of Bucharest. Two yeast strains were also tested: *Candida albicans* ATCC 10231 and *Candida parapsilosis* ATCC 22019 [53]. The CM and MFCM samples (10 mg) were placed in contact with a microbial suspension (10^4^ CFU/mL—final density) and were subjected to vigorous shaking in order to assure the best contact between microbial cells and the samples. At 0 h and 24 h contact times, the viable cell counts (CFU/mL) of the microbial suspension were determined by spotting serial 10-fold dilutions on Muller–Hinton agar for bacteria and Sabauroud for yeasts. The microbial lg reduction of microbial growth in the presence of the CM and MFCM samples was calculated as follows: recovery rate = lg (CFU sample)/lg (CFU control strain). 

### 4.5. Assessment of Cell Viability and Cytotoxicity by MTT and LDH Assay

MTT assay is a spectrophotometric method to analyze both cell viability and proliferation of L929 murine fibroblasts, which were co-cultivated with the CM and MFCM (at various concentrations: 100, 200, and 500 µg/µL) for 24 h and further incubated with 1 mg/mL MTT solution in the dark at 37 °C. After 4 h of incubation, formazan crystals were solubilized with isopropanol, resulting in a purple solution, and quantified at 550 nm using FlexStation 3 (Molecular Devices Company, Sunnyvale, CA, USA).

The level of cytotoxicity exerted on the L929 cells was quantified using the LDH test (Tox7 kit, Sigma-Aldrich, Burlington, MA, USA), following the manufacturer’s instructions [54].

### 4.6. Statistical Analysis

All the analyses were performed in triplicate. The statistical analysis was performed by one-way analysis of variance (one-way ANOVA) using Microsoft Office Excel 365 (Redmond, WA, USA) at a 95% confidence interval.

## 5. Conclusions

Here, the microcellulose obtained from *G. triacanthos* pods is, to our knowledge, reported for the first time. The cellulose was extracted from *G. triacanthos* pods and submitted to six cycles of delignification for purification. The extract from the *G. triacanthos* leaves used for the functionalization of the cellulose microfibers had a content of TPC 113.62 ± 1.09 mg GAE/g dry plant, with the major compounds identified by UHPLC–DAD-ESI/MS being catechin (34,271.15 µg/L), chlorogenic acid (11,984.43 µg/L), quercetin (3007.89 µg/L), cinnamic acid (2736.42 µg/L), epicatechin (1431.47 µg/L), rutin (1302.31 µg/L), and vanillic acid (773.88 µg/L).

The multi-functionalized cellulose microfibers with the phenolic compounds extracted from the leaves of the same species manifested improved absorptivity and fluid retention properties and assured the controlled release of the active principles. The antioxidant effect correlated with the antimicrobial activity revealed that MFCM may represent a promising biocompatibility material for the design of high-performance wound dressings that could prevent wound infection and facilitate the wound healing process. 

## Figures and Tables

**Table 1:** Recovery rate of logarithmical colony forming unit/mL (lg CFU/mL) compared to microbial strain control (the closer it is to 1, the lower the antimicrobial effect).

Strains	Time	CM	MFCM
*Staphylococcus aureus* MRSA clinical strain	0	0.997 ± 0.045	0.995 ± 0.026
	24	0.522 ± 0.021	0.513 ± 0.005
*Enterococcus faecium* ATCC DMS 13590	0	0.778 ± 0.014	0.752 ± 0.029
	24	0.744 ± 0.009	0.747 ± 0.029
*Pseudomonas aeruginosa* 6 clinical strain	0	0.792 ± 0.085	0.681 ± 0.098
	24	0.690 ± 0.075	0.591 ± 0.075
*Pseudomonas aeruginosa* ATCC 27853	0	0.933 ± 0.077	0.725 ± 0.028
	24	0.848 ± 0.172	0.701 ± 0.172
*Enterobacter cloacae* clinical strain	0	0.983 ± 0.016	0.987 ± 0.016
	24	0.821 ± 0.003	0.732 ± 0.005
*Acinetobacter baumannii* clinical strain	0	0.890 ± 0.011	0.784 ± 0.021
	24	0.723 ± 0.004	0.652 ± 0.011
*Escherichia coli* ATCC 11229	0	0.761 ± 0.032	0.708 ± 0.035
	24	0.894 ± 0.006	0.802 ± 0.051
*Candida albicans* ATCC 10231	0	0.987 ± 0.035	0.969 ± 0.007
	24	0.875 ± 0.006	0.776 ± 0.141
*Candida parapsilosis* ATCC 22019	0	0.996 ± 0.021	0.996 ± 0.024
	24	0.807 ± 0.01	0.732 ± 0.022

MRSA: Methicillin-Resistant *Staphylococcus aureus*, ATCC: American Type Culture Collection.

## Data Availability

Not Available.

## Supplementary Materials

The following are available online at https://www.mdpi.com/1422-0067/22/1/33/s1.

## Abbreviations

ABTS	2:2′-azinobis (3-ethylbenzothiazoline-6-sulfonic acid)
ATCC	American Type Culture Collection
CA	Contact Angle
CFU	Colony Forming Unit
CM	Cellulose Microfibers
DMSO	Dimethylsulfoxide
DPPH	1,1-diphenyl-2-picrylhydrazyl radical
ECB	Ethanol-Chlorobenzene
MFCM	Multi-functionalized cellulose microfibers (with a phenolic compound extract from *G. triachantos* leaves)
FTIR	Fourier Transform Infrared
ATR-FTIR	Attenuated Total Reflectance—Fourier Transform Infrared
GAE	Gallic acid equivalents
LDH	Lactate dehydrogenase
MF	Microcellulose fibers
MRSA	Methicillin-Resistant *Staphylococcus aureus*
MTT	[3-(4,5-dimethylthiazol-2yl)]-2,5-diphenyltetrazolium bromide
PAs	Phenolic acids
PBS	Phosphate-buffered saline
QE	Quercetin equivalents
ROS	Reactive oxygen species
SEM	Scanning electron microscopy
TE	Trolox equivalents
TEAC	Trolox equivalent antioxidant capacity
TEMPO	(2,2,6,6-tetramethyl-1-piperidinyl)oxidanyl
TPC	Total phenolic compounds
UHPLC–DAD-ESI/MS	Ultra-high-performance liquid chromatography diode array detector electrospray ionization tandem mass spectrometry

## References

[1] Bacakova L., Pajorova J., Bacakova M., Skogberg A., Kallio P., Kolarova K., Svorcik V. (2019). Versatile Application of Nanocellulose: From Industry to Skin Tissue Engineering and Wound Healing. Nanomaterials.

[2] Nie K., Song Y., Liu S., Han G., Ben H., Ragauskas A.J., Jiang W. (2019). Preparation and Characterization of Microcellulose and Nanocellulose Fibers from Artemisia Vulgaris Bast. Polymers.

[3] Sun F., Nordli H.R., Pukstad B., Kristofer Gamstedt E., Chinga-Carrasco G. (2017). Mechanical characteristics of nanocellulose-PEG bionanocomposite wound dressings in wet conditions. J. Mech. Behav. Biomed. Mater..

[4] Xie J., Lia J. (2017). Smart drug delivery system based on nanocelluloses. J. Bioresour. Bioprod..

[5] Denet A.-R., Ucakar B., Préat V. (2003). Transdermal Delivery of Timolol and Atenolol Using Electroporation and Iontophoresis in Combination: A Mechanistic Approach. Pharm. Res..

[6] Rasheed M., Jawaid M., Karim Z., Abdullah L.C. (2020). Morphological, Physiochemical and Thermal Properties of Microcrystalline Cellulose (MCC) Extracted from Bamboo Fiber. Molecules.

[7] Elanthikkal S., Gopalakrishnapanicker U., Varghese S., Guthrie J.T. (2010). Cellulose microfibres produced from banana plant wastes: Isolation and characterization. Carbohydr. Polym..

[8] Ferus P., Barta M., Konôpková J., Turčeková S., Maňka P., Bibeň T. (2013). Diversity in honey locust (*Gleditsia triacanthos* L.) seed traits across Danube basin. Folia Oecologica.

[9] Ferreras A.E., Funes G., Galetto L. (2015). The role of seed germination in the invasion process of Honey locust (*Gleditsia triacanthos* L., *Fabaceae*): Comparison with a native confamilial. Plant Species Biol..

[10] Ferreras A.E., Galetto L. (2010). From seed production to seedling establishment: Important steps in an invasive process. Acta Oecologica.

[11] Svečnjak L., Marijanović Z., Okińczyc P., Marek Kuś P., Jerković I. (2020). Mediterranean Propolis from the Adriatic Sea Islands as a Source of Natural Antioxidants: Comprehensive Chemical Biodiversity Determined by GC-MS, FTIR-ATR, UHPLC-DAD-QqTOF-MS, DPPH and FRAP Assay. Antioxidants.

[12] Danial W.H., Abdul Majid Z., Mohd Muhid M.N., Triwahyono S., Bakar M.B., Ramli Z. (2015). The reuse of wastepaper for the extraction of cellulose nanocrystals. Carbohydr. Polym..

[13] Garside P., Wyeth P. (2003). Identification of Cellulosic Fibres by FTIR Spectroscopy—Thread and Single Fibre Analysis by Attenuated Total Reflectance. Stud. Conserv..

[14] Edelmann A., Diewok J., Schuster K.C., Lendl B. (2001). Rapid Method for the Discrimination of Red Wine Cultivars Based on Mid-Infrared Spectroscopy of Phenolic Wine Extracts. J. Agric. Food Chem..

[15] Fernández K., Agosin E. (2007). Quantitative Analysis of Red Wine Tannins Using Fourier-Transform Mid-Infrared Spectrometry. J. Agric. Food Chem..

[16] Laghi L., Versari A., Parpinello G.P., Nakaji D.Y., Boulton R.B. (2011). FTIR Spectroscopy and Direct Orthogonal Signal Correction Preprocessing Applied to Selected Phenolic Compounds in Red Wines. Food Anal. Methods.

[17] Espinosa-Acosta G., Ramos-Jacques A., Molina G., Maya-Cornejo J., Esparza R., Hernandez-Martinez A., Sánchez-González I., Estevez M. (2018). Stability Analysis of Anthocyanins Using Alcoholic Extracts from Black Carrot (*Daucus Carota* ssp. *Sativus* Var. *Atrorubens* Alef.). Molecules.

[18] Qian S., Fang X., Dan D., Diao E., Lu Z. (2018). Ultrasonic-assisted enzymatic extraction of a water soluble polysaccharide from dragon fruit peel and its antioxidant activity. RSC Adv..

[19] Santos D.I., Neiva Correia M.J., Mateus M.M., Saraiva J.A., Vicente A.A., Moldão M. (2019). Fourier Transform Infrared (FT-IR) Spectroscopy as a Possible Rapid Tool to Evaluate Abiotic Stress Effects on Pineapple By-Products. Appl. Sci..

[20] Barbehenn R., Cheek S., Gasperut A., Lister E., Maben R. (2005). Phenolic Compounds in Red Oak and Sugar Maple Leaves Have Prooxidant Activities in the Midgut Fluids of *Malacosoma disstria* and *Orgyia leucostigma* Caterpillars. J. Chem. Ecol..

[21] Shetty B.S. (2013). Wound Healing and Indigenous Drugs: Role as Antioxidants: A Review. Res. Rev. J. Med. Heal. Sci..

[22] Fikru A., Makonnen E., Eguale T., Debella A., Abie Mekonnen G. (2012). Evaluation of in vivo wound healing activity of methanol extract of *Achyranthes aspera* L.. J. Ethnopharmacol..

[23] Mohammed R.S., Abou Zeid A.H., El Hawary S.S., Sleem A.A., Ashour W.E. (2014). Flavonoid constituents, cytotoxic and antioxidant activities of *Gleditsia triacanthos* L. leaves. Saudi J. Biol. Sci..

[24] Ham J.R., Lee H.-I., Choi R.-Y., Sim M.-O., Seo K.-I., Lee M.-K. (2016). Anti-steatotic and anti-inflammatory roles of syringic acid in high-fat diet-induced obese mice. Food Funct..

[25] Karimi-Khouzani O., Heidarian E., Amini S.A. (2017). Anti-inflammatory and ameliorative effects of gallic acid on fluoxetine-induced oxidative stress and liver damage in rats. Pharmacol. Rep..

[26] Pei K., Ou J., Huang J., Ou S. (2016). p -Coumaric acid and its conjugates: Dietary sources, pharmacokinetic properties and biological activities. J. Sci. Food Agric..

[27] Che D.N., Cho B.O., Shin J.Y., Kang H.J., Kim J.-S., Oh H., Kim Y.-S., Jang S. (2019). Il Apigenin Inhibits IL-31 Cytokine in Human Mast Cell and Mouse Skin Tissues. Molecules.

[28] Xiong G., Ji W., Wang F., Zhang F., Xue P., Cheng M., Sun Y., Wang X., Zhang T. (2019). Quercetin Inhibits Inflammatory Response Induced by LPS from *Porphyromonas gingivalis* in Human Gingival Fibroblasts via Suppressing NF- κ B Signaling Pathway. BioMed Res. Int..

[29] Semwal D., Semwal R., Combrinck S., Viljoen A. (2016). Myricetin: A Dietary Molecule with Diverse Biological Activities. Nutrients.

[30] Liu J., Du C., Beaman H.T., Monroe M.B.B. (2020). Characterization of Phenolic Acid Antimicrobial and Antioxidant Structure–Property Relationships. Pharmaceutics.

[31] Saleh D.O., Kassem I., Melek F.R. (2016). Analgesic activity of *Gleditsia triacanthos* methanolic fruit extract and its saponin-containing fraction. Pharm Biol..

[32] Xu W., Liu Y., Zhang F., Lei F., Wang K., Jiang J. (2020). Physicochemical characterization of Gleditsia triacanthos galactomannan during deposition and maturation. Int. J. Biol. Macromol..

[33] Zhuo X., Liu C., Pan R., Dong X., Li Y. (2017). Nanocellulose Mechanically Isolated from Amorpha fruticosa Linn. ACS Sustain. Chem. Eng..

[34] Khalil H.P.S.A., Jummaat F., Yahya E.B., Olaiya N.G., Adnan A.S., Abdat M., N. A. M. N., Halim A.S., Kumar U.S.U., Bairwan R. (2020). A Review on Micro- to Nanocellulose Biopolymer Scaffold Forming for Tissue Engineering Applications. Polymers.

[35] Yamane C., Aoyagi T., Ago M., Ago M., Sato K., Okajima K., Takahashi T. (2006). Two Different Surface Properties of Regenerated Cellulose due to Structural Anisotropy. Polym. J..

[36] Ngadaonye J.I., Geever L.M., Killion J., Higginbotham C.L. (2013). Development of novel chitosan-poly(N,N-diethylacrylamide) IPN films for potential wound dressing and biomedical applications. J. Polym. Res..

[37] Kickhöfen B., Wokalek H., Scheel D., Ruh H. (1986). Chemical and physical properties of a hydrogel wound dressing. Biomaterials.

[38] Schafer M., Werner S. (2008). Oxidative stress in normal and impaired wound repair. Pharmacol. Res..

[39] Działo M., Mierziak J., Korzun U., Preisner M., Szopa J., Kulma A. (2016). The Potential of Plant Phenolics in Prevention and Therapy of Skin Disorders. Int. J. Mol. Sci..

[40] Nascimento G.G.F., Locatelli J., Freitas P.C., Silva G.L. (2000). Antibacterial activity of plant extracts and phytochemicals on antibiotic-resistant bacteria. Brazilian J. Microbiol..

[41] Salaheen S., Peng M., Joo J., Teramoto H., Biswas D. (2017). Eradication and Sensitization of Methicillin Resistant *Staphylococcus aureus* to Methicillin with Bioactive Extracts of Berry Pomace. Front. Microbiol..

[42] Alves M.J., Ferreira I.C.F.R., Froufe H.J.C., Abreu R.M.V., Martins A., Pintado M. (2013). Antimicrobial activity of phenolic compounds identified in wild mushrooms, SAR analysis and docking studies. J. Appl. Microbiol..

[43] Merkl R., Hrádková I., Filip V., Šmidrkal J. (2010). Antimicrobial and antioxidant properties of phenolic acids alkyl esters. Czech J. Food Sci..

[44] Chatterjee N.S., Panda S.K., Navitha M., Asha K.K., Anandan R., Mathew S. (2015). Vanillic acid and coumaric acid grafted chitosan derivatives: Improved grafting ratio and potential application in functional food. J. Food Sci. Technol..

[45] Singleton V., Rossi J. (1965). Colorimetry of Total Phenolics with Phosphomolybdic-Phosphotungstic Acid Reagents. Am. J. Enol. Vitic..

[46] Woisky R.G., Salatino A. (1998). Analysis of propolis: Some parameters and procedures for chemical quality control. J. Apic. Res..

[47] Ciucure C.T., Geană E. (2019). Phenolic compounds profile and biochemical properties of honeys in relationship to the honey floral sources. Phytochem. Anal..

[48] Gaur R., Azizi M., Gan J., Hansal P., Harper K., Mannan R., Panchal A., Patel K., Patel M., Patel N. (2009). British Pharmacopoeia.

[49] Stan G.E., Tite T., Popa A.-C., Chirica I.M., Negrila C.C., Besleaga C., Zgura I., Sergentu A.C., Popescu-Pelin G., Cristea D. (2020). The Beneficial Mechanical and Biological Outcomes of Thin Copper-Gallium Doped Silica-Rich Bio-Active Glass Implant-Type Coatings. Coatings.

[50] Lu B., Wang T., Li Z., Dai F., Lv L., Tang F., Yu K., Liu J., Lan G. (2016). Healing of skin wounds with a chitosan–gelatin sponge loaded with tannins and platelet-rich plasma. Int. J. Biol. Macromol..

[51] Madhu G., Bose V.C., Aiswaryaraj A.S., Maniammal K., Biju V. (2013). Defect dependent antioxidant activity of nanostructured nickel oxide synthesized through a novel chemical method. Colloids Surfaces A Physicochem. Eng. Asp..

[52] Re R., Pellegrini N., Proteggente A., Pannala A., Yang M., Rice-Evans C. (1999). Antioxidant activity applying an improved ABTS radical cation decolorization assay. Free Radic. Biol. Med..

[53] Ong S.Y., Wu J., Moochhala S.M., Tan M.H., Lu J. (2008). Development of a chitosan-based wound dressing with improved hemostatic and antimicrobial properties. Biomaterials.

[54] Serbezeanu D., Vlad-Bubulac T., Rusu D., Grădișteanu Pircalabioru G., Samoilă I., Dinescu S., Aflori M. (2019). Functional Polyimide-Based Electrospun Fibers for Biomedical Application. Material.

