# Supplementary material for: Valorization of Gleditsia triacanthos Invasive Plant Cellulose Microfibers and Phenolic Compounds for Obtaining Multi-Functional Wound Dressings with Antimicrobial and Antioxidant Properties
Source: Int J Mol Sci. 2020 Dec 22;22(1):33. doi: 10.3390/ijms22010033 (PMC7792949; doi:10.3390/ijms22010033)
Supplement: Supplementary file 1 [file ijms-22-00033-s001.pdf]

International Journal of
Molecular Sciences

MDPI

Table S1. The UHPLC-MS/MS identification and quantification data of phenolic acids (PAs) and flavonoids in the alcoholic extract of *Gleditsia triachantos*

Compound Name	Exact Mass[a] [M-H]−	Accurate Mass [M-H]−	Mass Fragments (Intensity, %)	Collision Energy (eV)	Concentration (μg/L)
			**Phenolic Acids (PA)**		
4-hydroxy benzoic acid	137.0243	137.0232	93.0331	30	570.37
vanillic acid	167.0349	167.0343	152.0105, 124.0154, 111.0075, 139.0025, 95.0125	60	773.88
syringic acid	197.0455	197.0450	182.0212, 166.9976, 153.0547, 138.0311, 123.0075	30	163.71
protocatechuic acid	153.0188	153.0183	109.0281	35	235.61
gallic acid	169.0142	169.0133	125.0231	35	86.28
p-coumaric acid	163.0400	163.0392	119.0489	35	192.96
ferulic acid	193.0506	193.0500	178.0262, 134.0361	30	319.81
caffeic acid	179.0349	179.0342	135.044	30	153.97
			**Flavonoids**		
catechin	289.0717	289.0719	109.0282, 125.0232,	35	34,271.15
epicatechin	289.0717	289.0719	137.0232, 151.0390, 203.0708, 245.0817		1,431.47
pinocembrin	255.0662	255.0663	213.0551, 151.0026, 107.0125	40	11.34
pinostrobin	269.0819	269.0822	179.0554,	45	602.94
crysin	253.0506	253.0506	143.0491, 145.0284, 107.0125, 209.0603, 63.0226, 65.0019	60	21.52
apigenin	269.0455	269.0457	117.0333, 151.0027, 107.0126	60	676.17
quercetin	301.0353	301.0356	151.0226, 178.9977, 121.0282, 107.0125	35	3,007.89
isorhamnetin	315.0509	315.0512	300.0276	45	228.61
kaempferol	285.0404	285.0406	285.0405	45	N.d.
myricetin	317.0302	317.0306	289.0720, 179.0342	30	1,259.97
galangin	269.0455	269.0458	169.0650, 143.0491,	80	2.51
rutin	609.1460	609.1469	420.1880, 187.0969	30	1,302.31
hesperidin	609.1824	609.1823	301.0171	30	13.07
naringin	579.1718	579.1720	271.0612, 459.1133, 151.0026	30	168.63

[a] Calculated mass of the parent ion using free chemical database, ChemSpider; n.d.-not detected.
